# Miniaturizable Ion-Selective Arrays Based on Highly Stable Polymer Membranes for Biomedical Applications

**DOI:** 10.3390/s140711844

**Published:** 2014-07-04

**Authors:** Mònica Mir, Roberto Lugo, Islam Bogachan Tahirbegi, Josep Samitier

**Affiliations:** 1 Nanobioengineering Laboratory, Institute for Bioengineering of Catalonia (IBEC), Baldiri Reixac, 10–12, Barcelona 08028, Spain; E-Mails: ibq_robertolugo@hotmail.com (R.L.); ibogachan@ibecbarcelona.eu (I.B.T.); jsamitier@ibecbarcelona.eu (J.S.); 2 Centro de Investigación Biomédica en Red de Bioingeniería, Biomateriales y Nanomedicina (CIBER-BBN), Maria de Luna, 11, Zaragoza 50018, Spain; 3 Department of Electronics, Barcelona University (UB), Martí I Franques, 1, Barcelona 08028, Spain

**Keywords:** ion-selective electrode (ISE) sensor, pH detection, ischemia, electrochemistry, implantable device, biomedicine, endoscope

## Abstract

Poly(vinylchloride) (PVC) is the most common polymer matrix used in the fabrication of ion-selective electrodes (ISEs). However, the surfaces of PVC-based sensors have been reported to show membrane instability. In an attempt to overcome this limitation, here we developed two alternative methods for the preparation of highly stable and robust ion-selective sensors. These platforms are based on the selective electropolymerization of poly(3,4-ethylenedioxythiophene) (PEDOT), where the sulfur atoms contained in the polymer covalently interact with the gold electrode, also permitting controlled selective attachment on a miniaturized electrode in an array format. This platform sensor was improved with the crosslinking of the membrane compounds with poly(ethyleneglycol) diglycidyl ether (PEG), thus also increasing the biocompatibility of the sensor. The resulting ISE membranes showed faster signal stabilization of the sensor response compared with that of the PVC matrix and also better reproducibility and stability, thus making these platforms highly suitable candidates for the manufacture of robust implantable sensors.

## 1. Introduction

Glass electrodes are the most popular electrodes used for the determination of pH because of their high selectivity, trustworthiness and pH dynamic range. These sensors are fabricated with an electrode in contact with an inner solution held by a selective glass membrane. Although pH-sensitive glass electrodes are widely used in many applications, they have certain drawbacks, such as high electric resistance, and instability in hydrofluoric acid, fluoride and silane solutions. However, the most critical limitations of glass-based pH sensor are their fragility and the difficulty to achieve miniaturization, two factors that restrict their applicability, especially as implantable sensors.

The last two decades have witnessed a breakthrough in the field that overcomes this limitation, namely the development of solid sensors for pH detection [1]. Solid ion-selective electrode (ISE) membranes have become a routine tool for the electroanalysis of diverse kinds of samples since they significantly improve the analytical parameters and are more resistant and easy to miniaturize. Potentiometry is the electrochemical technique most commonly used in the readout of solid ISE sensors. This technique is especially attractive because of its high sensitivity, low levels of detection and low cost [2]. The selectivity of the sensors to certain ions is achieved by means of a selective polymeric membrane immobilized on the sensor surface, which holds a chemical receptor called ionophore, additives that improve the selectivity of the membrane and reduce its electrical resistance, and a plasticizer that attaches the ionophore and additives on the sensor surface. The ion-selective membrane should be chemically stable and inert and should show low electrical resistance, biocompatibility and non-toxicity [3]. Poly(vinyl chloride) (PVC) is the most commonly used polymer matrix in solid ISE sensors. However, this polymer shows poor adhesion to the surface of the transducer [4], mainly because the parameter governing the immobilization of the PVC membranes is the adsorption through weak Van de Waals interactions. It is crucial to achieve strong membrane attachment to the surface of the sensor, as a weak link can lead to serious problems regarding the stability and reproducibility of the response [5]. Fragmentation or leaching of membrane compounds may cause shortened sensor lifetime and loss of functionality. Leaching studies on ISE sensors based on covalently linked benzo-18-crown-6 with multi-wall carbon nanotubes were performed by Parra *et al.* [6]. However, even greater are the drawbacks with regard to toxicity [7,8], in particular when these compounds are not hemocompatible, as is the case of PVC [9]. Moreover, blood fouling on the PVC membrane leads to rejection and passivation of the sensor [10]. In terms of electrical signal transfer, thick PVC layers hinder the transport of electrochemical signals and ion diffusion through the membrane. The thermal stability of PVC membranes is also too low for the purposes of several applications [11].

**Figure 1.**
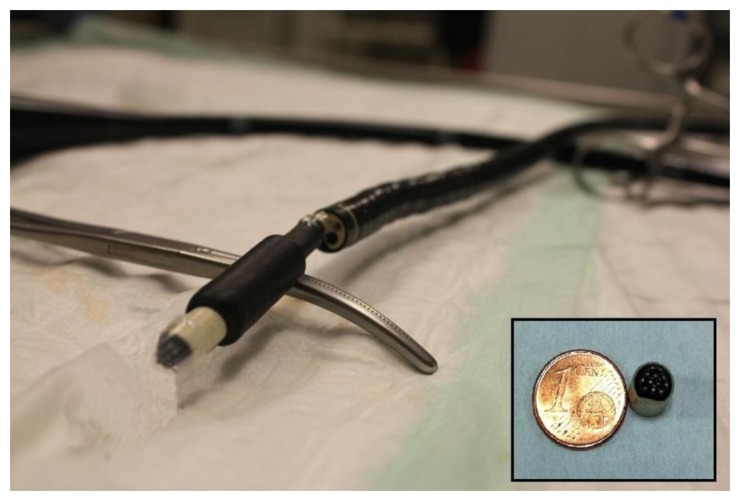
Picture of the developed sensor array inserted in a gastroendoscope. The inlet picture shows the size of the array.

In order to overcome the problems concerning PVC membranes, here we developed two ISE sensor platforms for sensing pH. Both devices were designed to be used with endoscopic systems. Since the final application of the sensor addresses *in vivo* detection inside the body, we devoted special attention to membrane stability and leaching to obtain a harmless platform. Moreover, the strong acidic and corrosive conditions found in the stomach tissue require a stable sensor that could bear these conditions. The two sensor configurations were based on the electropolymerization of poly(3,4-ethylenedioxythiophene) (PEDOT) on the electrode surface with the ionophore and additives entrapped in the matrix. Electropolymerization was achieved by applying a voltage in the electrode that we wished to be functionalized, while the other electrodes inside the same solution were not affected. This technique allows the selective fabrication of the ISE on a specific and miniaturized sensor in an array format [12], whereas the manual deposition of the traditional PVC membranes does not permit the correct functionalization of nano/microsystems. PEDOT, which contains sulfur groups, binds strongly with the gold surface through dative binding [13], thereby conferring a highly stable conformation, which in turn impedes leaching and degradation of the membrane. Moreover, PEDOT is considered a conductive polymer that improves electrical signal transfer [14]. In order to covalently attach a certain concentration of all sensor compounds, a crosslinker, poly(ethylene glycol) diglycidyl ether (PEG), was mixed with PEDOT in the second configuration developed. The PEG layer also offers the advantages of flexibility and biocompatibility [15]. A prototype of the potentiometric 12-electrodes array containing the pH sensor developed here was fabricated in an appropriate size and shape for insertion into the body through a gastroendoscope. The device proposed is non-invasive, harmless, inexpensive, and portable, and it shows a rapid response. The array aims to monitor ischemia—an event related to a change in pH—*in vivo* in the stomach during surgical procedures performed with a laparoscopic teleoperated robot [16] (Figure 1).

## 2. Experimental Section

### 2.1. Materials

Tridodecylamine, potassium tetrakis(4-chlorophenyl)borate, 2-nitrophenyl octyl ether, high molecular weight poly(vinyl chloride), tris(hydroxymethyl)aminemethane (TRIS), tetrahydrofuran, poly(3,4-ethylenedioxythiophene) poly(styrenesulfonate), and poly(ethylene glycol) diglycidyl ether were purchased from Sigma-Aldrich (St. Louis, MO, USA).

### 2.2. Equipment

All the electrochemical techniques were performed with a CHI684C multipotentiostat from CH Instruments, Inc. (Austin, TX, USA). The thickness of the membranes fabricated for these experiments were measured with a Dektak 6M profilometer from Veeco (Plainview, NY, USA). The time-of-flight secondary ion mass spectrometry (TOF-SIMS) analyses were performed using a TOF-SIMS IV (ION-TOF, Munster, Germany) operating at 5 × 10^−9^ mbar. Samples were bombarded with a pulsed bismuth liquid metal ion source (Bi^3+^) at an energy of 25 keV. The gun was operated with a 20-ns pulse width, 0.3 pA pulsed ion current for a dosage lower than 5 × 10^11^ ions/cm^2^, well below the threshold level of 1 × 10^13^ ions/cm^2^ generally accepted for static SIMS conditions. Secondary ions were detected with a reflector time-of-flight analyzer, a multichannel plate, and a time-to-digital converter. Measurements were performed with a typical acquisition time of 10 s, at a TDC time resolution of 200 ps and 100 us cycle time. Charge neutralization was achieved with a low energy (20 eV) electron flood gun. Secondary ions were extracted with a voltage of 2 kV and were post-accelerated to a kinetic energy of 10 keV just before hitting the detector. Mass spectral acquisition was performed with the ION-TOF Ion Spec software (version 4.1). Each spectrum is normalized to total intensity.

### 2.3. Methods

#### 2.3.1. Electrode Cleaning

The electrode arrays were cleaned electrochemically in 0.5 M H_2_SO_4_ by scanning the potential between the oxidation and reduction of gold, −0.05 V and +1.2 V *versus* Ag/AgCl reference electrode, until there was no further change in the voltammogram. In each cycle a monolayer of chemisorbed oxygen is formed and reduced, thus oxidizing all the organic material on the surface.

#### 2.3.2. ISE Performed with PVC Membranes

A protocol from Sigma-Aldrich was followed for the fabrication of the commercial PVC ISE membrane. This protocol involved mixing four compounds in the following proportion: 1.00% wt tridodecylamine, 0.60% wt 4-chlorophenyl borate, 66.00% wt 2-nitrophentyl-octyl-ether and 32.40% wt poly(vinyl chloride); all dissolved in tetrahydrofuran (THF). Two μL of this membrane mixture was deposited on the gold electrode surface and was left to dry for 24 h.

#### 2.3.3. ISE Performed with PEDOT Membranes

PEDOT (1.6 mM) and LiClO_4_ (0.1 M) in acetonitrile were mixed with 1% wt tridodecylamine, 0.60% wt potassium tetrakis(4-chlorophenyl)borate, and 66.0% wt 2-nitrophenyl octyl ether. This mixture was electropolymerized on the electrode surface by means of cyclic voltamperometry (CV), using Ag/AgCl as reference and platinum counter electrodes. The CV was run from −0.4 V to 1.2 V continuously until stable CVs were recorded. The unattached polymer was washed out with double deionized water and the electrode was left to dry.

#### 2.3.4. ISE Performed with PEDOT-PEG Membranes

For the preparation of these membranes, we followed the same protocol as that used for the PEDOT membranes, but 10% PEG (2 μL) was added after electropolymerization. The membrane was then left to crosslink overnight.

#### 2.3.5. Potentiometric pH Detection

Once the working electrode had been functionalized with the corresponding ion-selective membrane, it was used to sense the potentiometry pH changes in a solution. The electrochemical cell consisted of the modified gold working electrode and an Ag/AgCl reference electrode. We then added 50 mM TRIS to the electrochemical cell at pH 10 and injected volumes of a range of HCl concentrations in order to gradually change the pH.

## 3. Results and Discussion

With the aim of addressing the stability limitations of PVC-based ISEs, we developed two alternative ISE sensor configurations and compared them with PVC membranes. One system was based on the electropolymerization of PEDOT on the electrode surface. This polymer self-assembles on a monolayer format on the sensor, entrapping the rest of the membrane components that are in the solution during the polymerization, and the sulfur group in the polymer strongly attaches the complex through dative binding with the gold. Also, to achieve covalent attachment of the rest of the membrane components to each other and to the PEDOT polymer on the surface, we mixed a PEG polymer with the previous system. This polymer contains highly reactive epoxy cycles that interact with many kinds of functional groups, thus crosslinking all the compounds in the matrix. This interaction provides strong and stable attachment of all the membrane molecules with the sensor surface. Moreover, the use of PEG polymers as anti-fouling material is widely reported for improving the biocompatibility of surfaces [17]. The use of these polymers in this sensor platform conferred a major advantage for its application *in vivo.*

**Figure 2.**
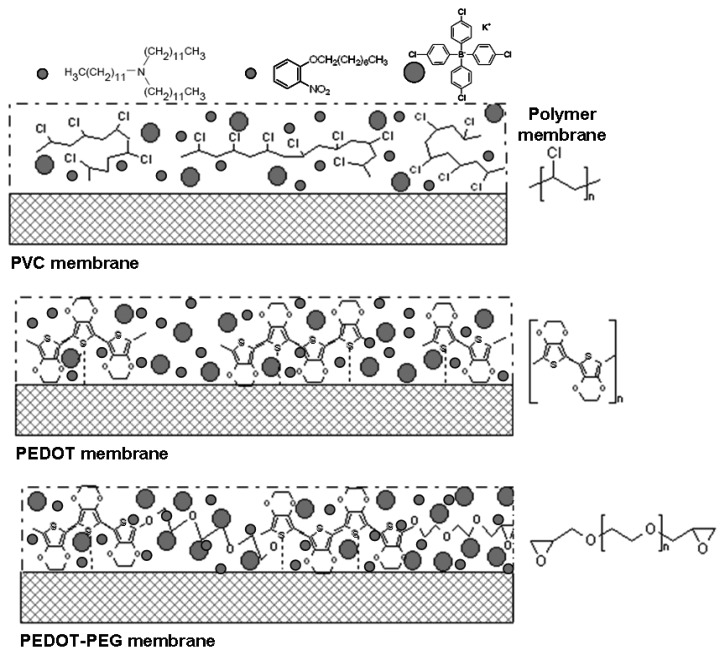
Schematic drawing of the ion-selective membranes tested and compared.

Figure 2 shows a simplified image of the three ion-selective membranes described above. The three sensor configurations were fabricated on the same kind of electrodes and measured in the same way. These membranes were tested and compared with potentiometric measurements of pH.

**Figure 3.**
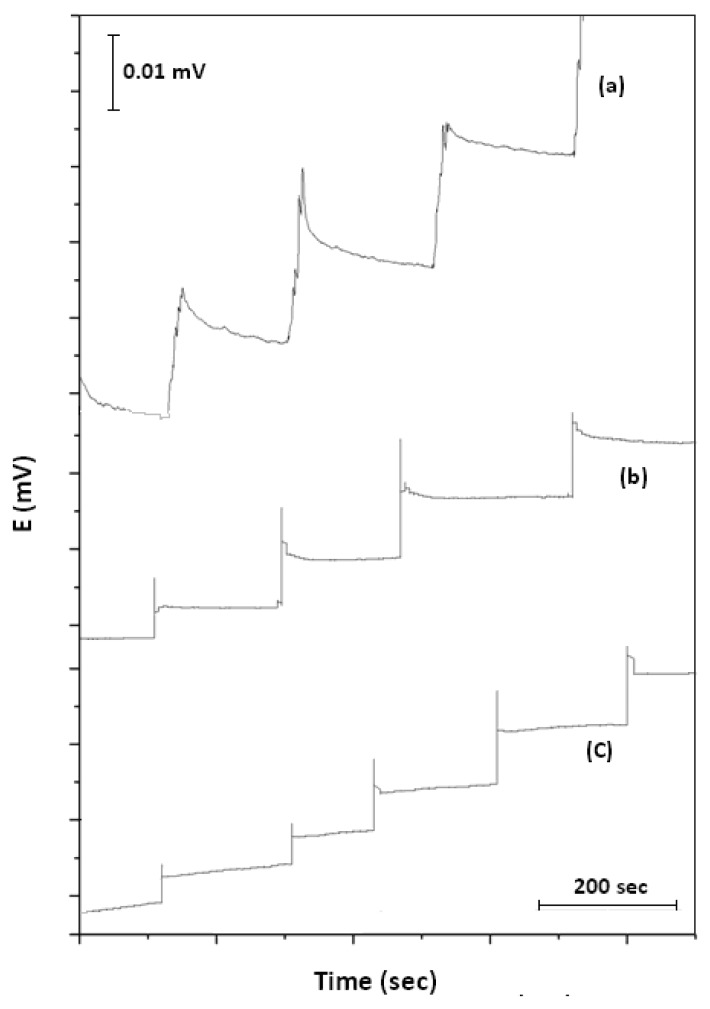
Kinetic response of the three ion-selective membranes tested; (**a**) PVC; (**b**) PEG; and (**c**) PEDOT after HCl injections.

Similar pH response curves were observed for the three systems, but with some relevant differences. The PEDOT membrane required little time to be stabilized at a low concentration of acid. The PVC sensor had a thicker membrane (20 μm), and it was not distributed uniformly on the electrode surface, compared with the homogeneous 7-nm monolayer of PEDOT membranes. The greater thickness of the flexible PVC membrane increased the instability of the sensor response. However, any perturbation of the system was translated and registered in the PVC sensor as a noisy and fluctuating response, as can be appreciated in Figure 3a. On the other hand, PEDOT membranes showed a stable response after applying the same perturbations (the injection of the acid) into the system (Figure 3b,c). Furthermore, the distinct diffusion of the ions into the thicker PVC membrane, compared with the PEDOT sensors, can be appreciated in this figure. The PVC sensor required a minimum of 200 s to reach a baseline response, while the PEDOT sensor took approximately 40 s. However, the PEDOT-PEG membrane showed better performance, achieving this response in about 10 s.

In terms of electrical signal transfer, the thicker PVC layer also hindered electrochemical signal transport. The bulk resistance of the membranes was measured with impedance spectroscopy. The resistance values were obtained by fitting the impedance response in an R_solution_ + R_membrane_/C_membrane_ circuit. The PVC membrane showed a resistance two orders of magnitude higher than that of the PEDOT membranes (1.3e^6^ Ω *vs.* 1.4e^4^ Ω, respectively).

**Figure 4.**
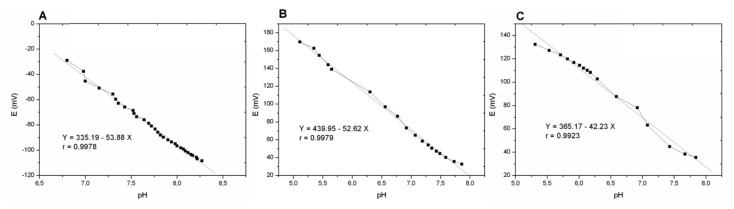
Potentiometric linear response *versus* pH of the three systems: (**A**) PVC; (**B**) PEDOT; and (**C**) PEG.

The potentiometric response caused by the changes in the pH solution was measured from pH 1 to 10, and the linear response of the membranes was plotted and compared to the Nernst equation:

$$E=E_{o}+2.303\left(RT/zF\right)\log\;a_{H+}$$

where *E* is the half-cell reduction potential, *E_o_* is the standard half-cell reduction potential, *R* is the universal gas constant, T is the absolute temperature, *z* is the number of electrons transferred in the cell reaction, *F* is the Faraday constant, and *a_H+_* is proton activity. Thus, by plotting *E versus* pH the slope of the fitted curve is related to *2.303RT/zF*, which in a monovalent reaction is equal to 59.5 mV. The linear fitting and the slope of each system is compared in Figure 4.

Few differences are observed on the slope of the linear response for the PVC commercial membrane and the PEDOT membrane, both exhibiting a near-Nernstian response (−53.88 and −52.62 mV/decade, respectively). In contrast, sub-Nernstian behavior (−42.23 mV/decade) was observed for sensors prepared with PEDOT-PEG membranes. The decrease in the response slope in hydrophilic and charge density surfaces, as in the case of this PEG-modified surface, has been reported on alkylsilane-modified potentiometric pH sensors [18,19]. As reported charged and conductive polymers have more difficulties to achieve a great selectivity in ISE membrane. However, this work is focused in the development of an ISE sensor that has to be inserted inside the body. So the essential issue was to assure a stability and harmlessness platform, being sensitive and selective enough for this specific application.

**Figure 5.**
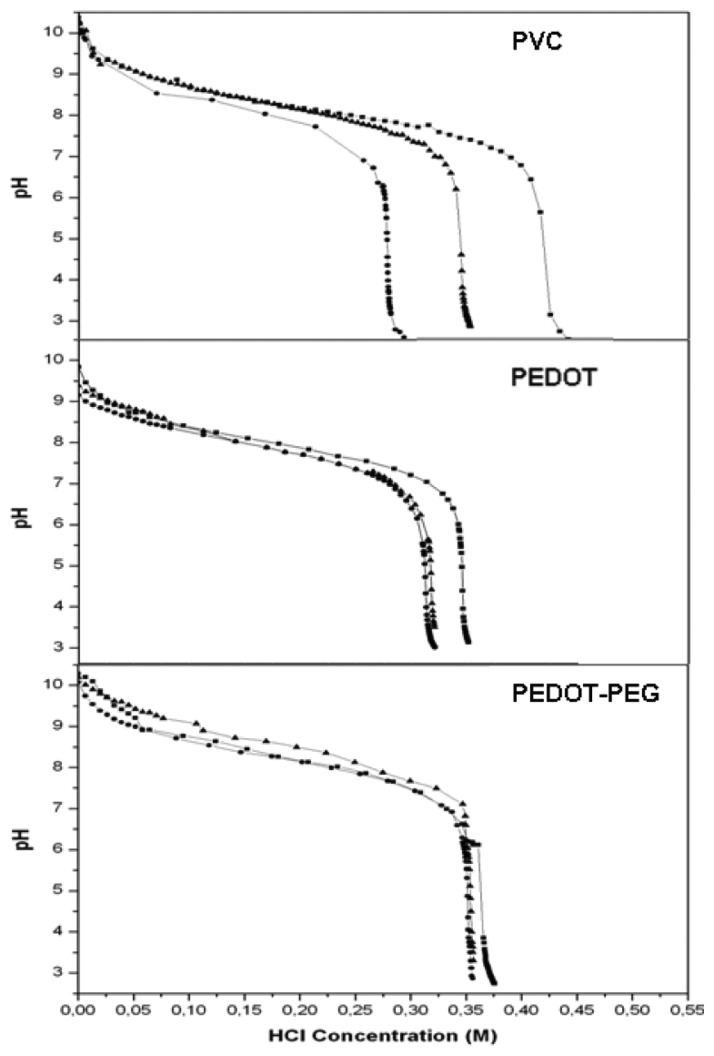
Repetitions (n = 3) of the potentiometric pH response for each sensor configuration.

We also tested the reproducibility of the systems. For this purpose, repetitions of each sensor platform was measured and compared in the same conditions. Better reproducibility of the response was recorded for the systems based on PEDOT and PEG (RSD = 0.019 and RSD = 0.009, respectively), compared with that based on PVC (RSD = 0.068), which was 7.5 times more irreproducible than the PEG configuration (Figure 5). The thick PVC membrane layer was distributed non-uniformly on the sensor surface. It also showed leakage of part of the membrane compounds, thus increasing the irreproducibility response of this kind of sensor. It has been reported that the formation of a thin aqueous layer between the gold metal sensor and the PVC ion-selective membranes caused by the instability and the weak interaction of this membrane on the sensor are responsible for potential drift of the sensor response and thus irreproducibility of response values [20].

We tested and compared the stability of the three ISE membranes on the gold electrode surface. In order to accelerate the bleeding of the ionophores and additives from the matrix and the fragmentation of the membrane, the three membranes were prepared on the same kind of gold electrodes and immersed in double deionized water under vigorous stirring conditions for 24 h. The electrodes were then dried with nitrogen, and the sensor surfaces were characterized by TOF-SIMS. This technique is based on bombardment of the sensor surface with an ion beam, in order to remove atoms, molecular fragments and ions from a depth of 10 nm of the membrane attached on the surface. These fragments are ejected into a mass spectrometer, where they are separated on the basis of their mass/charge ratio. The structure and composition of these fragments are directly related to the molecular structure of the surface.

**Figure 6.**
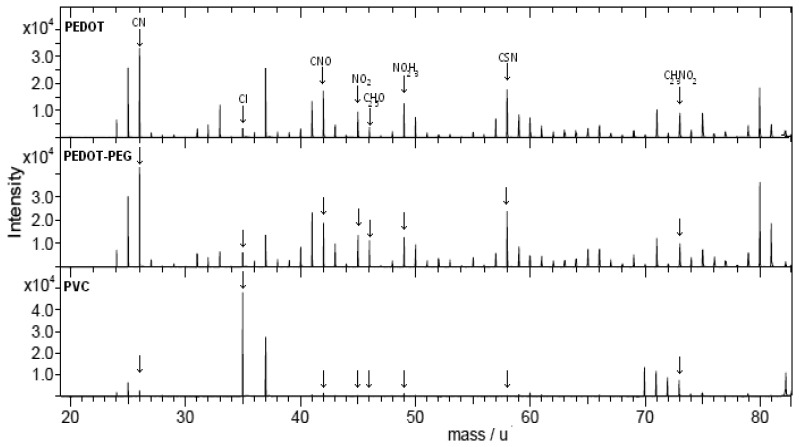
TOF-SIMS relative intensity spectra of the different isotopes on the PVC, PEDOT and PEDOT-PEG sensors.

The chemical structure of all the components of the ISE membranes is shown in Figure 2. The ionophore and the two additives were common in the three membranes and were immobilized in the same percentage. The molecule fragments specific to the additives and the ionophore were the following: CN, CNO, NO_2_, C_2_H_3_O, NO_2_H_2_, and C_2_HNO_2_. Thus, the qualitative study of the percentage of these ions in each membrane with TOF-SIMS, after the strong conditions applied in all the sensors, sheds light on the instability and leaching of the membrane compound in each sensor. The fragments produced after the electron beam shot on the three sensor membranes are shown in Figure 6.

We detected a similar relative intensity of the ions related with the ionophore and the additives in the PEDOT and PEDOT-PEG sensors. In contrast, these ions had low or negligible intensity in the PVC sensor. This observation reveals that after 24 h of strong stirring in double deionized water the PVC sensor showed more leaching of its compounds and that with the covalent binding of all their compounds the two PEDOT-based sensors achieved higher stability of membrane components.

## 4. Conclusions/Outlook

This study represents another step forward in efforts that started two decades ago to develop ion-selective sensors not based on glass. Here we have described new strategies for designing two novel ion-selective platforms that improve traditional membranes based on PVC, the most widely used matrix for this kind of sensor. Based on the electropolymerization of PEDOT and the crosslinking of PEG, these platforms offer a high stable conformation by means of the covalent bind of the polymer with the electrode, thereby avoiding the leaching and degradation of the membrane, as verified by TOF-SIMS results. Faster stabilization of the sensor response after injection of the analyte and better reproducibility was also demonstrated. Moreover, the electropolymerization technique has the advantage that it allows selective fabrication of the ISEs on a specific sensor in an array format, and the PEG layer confers flexibility and biocompatibility.

The results presented here demonstrate that these two PEDOT-based ISE sensors overcome the instability and irreproducibility problems reported for commercial PVC ISE membranes. Thus the features of the PEDOT-based ISE sensors makes them good candidates for endoscopic sensing in the stomach, as well as for other implantable device applications.
